# Antiviral Effect of Lithium Chloride on Replication of Marek’s Disease Virus in Chicken Embryonic Fibroblasts

**DOI:** 10.3390/ijms222212375

**Published:** 2021-11-16

**Authors:** Huifeng He, Dandan Qiao, Lu Zhang, Yongxiu Yao, Hongxia Shao, Aijian Qin, Kun Qian

**Affiliations:** 1 Ministry of Education Key Lab for Avian Preventive Medicine, Yangzhou University, No. 48 East Wenhui Road, Yangzhou 225009, China; hhf595115364@163.com (H.H.); zl13730687897@163.com (L.Z.); hxshao@yzu.edu.cn (H.S.); aijian@yzu.edu.cn (A.Q.); 2 Jiangsu Key Lab of Preventive Veterinary Medicine, Yangzhou University, No. 48 East Wenhui Road, Yangzhou 225009, China; qiaodandan20210603@163.com; 3 The Pirbright Institute & UK-China Centre of Excellence for Research on Avian Diseases, Pirbright, Surrey GU24 0NF, UK; yongxiu.yao@pirbright.ac.uk; 4 The International Joint Laboratory for Cooperation in Agriculture and Agricultural Product Safety, Ministry of Education, Yangzhou University, Yangzhou 225009, China

**Keywords:** Marek’s disease virus, lithium chloride, virus replication, inhibition

## Abstract

To investigate the antiviral effect of lithium chloride (LiCl) on the replication of Marek’s disease virus (MDV) in chicken embryonic fibroblast (CEF) cells, real-time PCR, Western blotting, plaque counting, and indirect immunofluorescence experiments were performed at different time points of LiCl treated CEF cells with virus infection. The results demonstrated that LiCl could affect multiple steps of virus replication and inhibit viral gene expression and protein synthesis in a dose- and time-dependent manner. Moreover, LiCl could directly affect viral infectivity as well. In addition, LiCl significantly affected the gene expression of IFN-β related genes in virus-infected cells. These results indicate that LiCl significantly inhibits MDV replication and proliferation in CEF cells and it has the potential to be used as an antiviral agent against MDV.

## 1. Introduction

Marek’s disease (MD) is a highly contagious disease characterized by the proliferation of T lymphoid tissue cells caused by Marek’s disease virus (MDV) belonging to the Family Herpesviridae [1]. MD is named after Jozsef Marek, a Hungarian veterinarian, who first reported the disease in 1907 [2]. MDV infection not only induces T cells tumors, but is also accompanied by significant immunosuppression and paralysis resulting in huge economic losses to the global poultry industry [1,3]. According to the published reports, most of the countries in the world have reported cases of MD [4]. Effective vaccination using live attenuated vaccines can prevent clinical MD, but has limited effect on virus replication and shedding [5,6]. This is thought to be one of the reasons for the continuous evolution of MDV virulence and emergence of hypervirulent pathotypes of the virus [7]. In recent years, there have been reports of the incidence of MD in the field in spite of vaccination [8,9]. Therefore, the study of antiviral compounds that have the potential for inhibiting MDV replication is beneficial for the control of MD.

Lithium chloride (LiCl) is a drug approved by the Food and Drug Administration (FDA), which is commonly used to treat patients with epilepsy and bipolar disorder [10,11,12]. Previous reports have shown that lithium can participate in a variety of life activities in cells, such as cell metabolism, intracellular material renewal, cell differentiation, migration, apoptosis, glycogen synthesis, gene expression, and inflammation [13]. Moreover, LiCl is also related to tumors, immune diseases, and nerves [10]. The earliest evidence that lithium had an effect on viral infection came from the antiviral effect of LiCl on herpes simplex virus (HSV) infection [14,15]. Later, an increasing number of studies showed that lithium salt treatment could reduce the production of multiple DNA or RNA virus infections, including pseudorabies virus (PRV) [16], canine parvovirus (CPV) [17], transmissible gastroenteritis virus (TGEV) [18], infectious bronchitis virus (IBV) [19], human immunodeficiency virus (HIV), and the avian leukosis virus subgroup J (ALV-J) virus previously reported by our group [20]. However, the effect of LiCl on the replication and proliferation of MDV has not been examined.

The purpose of the current study was to investigate the effect of LiCl treatment of CEF cells on the replication and proliferation of MDV. Our study showed that LiCl treatment can significantly inhibit the replication and proliferation of MDV, suggesting that LiCl has the potential to be used as an effective anti-MDV compound.

## 2. Results

### 2.1. LiCl Inhibits MDV Replication in CEF Cells

**Figure 1:**
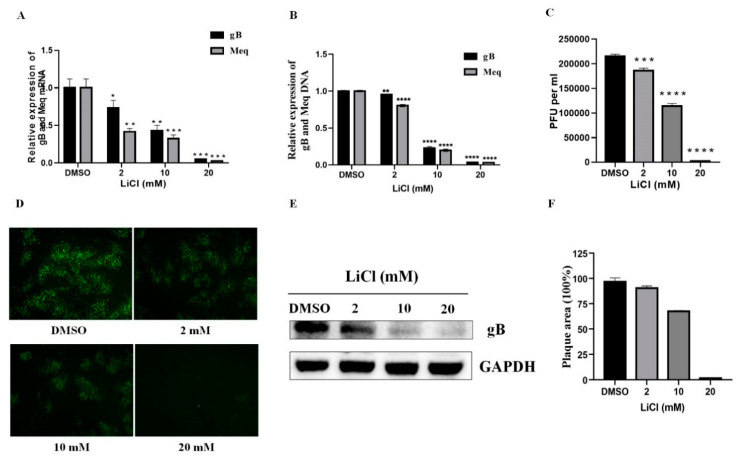
LiCl inhibits the replication of MDV in CEF. Detection of transcript level of gB and Meq genes (**A**) by qRT-PCR and MDV DNA quantity by qPCR (**B**) in CEF infected with MDV RB-1B strain after 96 h treatment with 0, 2, 10, and 20 mM LiCl. (**C**) Plaque count results of MDV RB-1B infected CEF treated with 0, 2, 10, and 20 mM LiCl. (**D**) Determination of the inhibitory effect of LiCl on MDV RB-1B plaque formation in CEF by indirect immunofluorescence assay. The pictures were captured with an OLYMPUS microscope (×200). (**E**) Detection of gB protein expression after treatment with 0, 2, 10, and 20 mM LiCl by Western blot using GAPDH as loading control. (**F**) Plaques were visualized with a fluorescence microscope. Plaque sizes (30 plaques) were measured, and presented as mean plaque sizes relative to the DMSO group. The mean plaque size of the DMSO group was set to 100%. The error bar represents the standard deviation of three independent experiments. The data are expressed as the average ± SD of three independent experiments, and analyzed by Student-t Test (* *p* < 0.05, ** *p* < 0.01, *** *p* < 0.001, **** *p* < 0.0001) for A, B, and C.

The previous cytotoxicity test of LiCl on CEF cells in our group revealed that the concentrations of 0, 2, 10, and 20 mM LiCl had no cytotoxicity on CEF cells [20]. After 96 h of inoculation with the MDV RB-1B strain, it was found that LiCl inhibited the transcription of MDV gB and Meq genes in a dose-dependent manner compared with the DMSO control group. The difference in transcription levels was most significant when the LiCl concentration was 20 mM (Figure 1A). Similar results were found in MDV DNA Quantity detected by qPCR (Figure 1B). The results of virus titration, indirect immunofluorescence staining, Western blot, and plaque area measurement demonstrated that LiCl treatment also significantly inhibited viral protein expression and plaque formation (Figure 1C–F).

### 2.2. Time-Dependent Manner of LiCl Inhibition on MDV Replication

**Figure 2:**
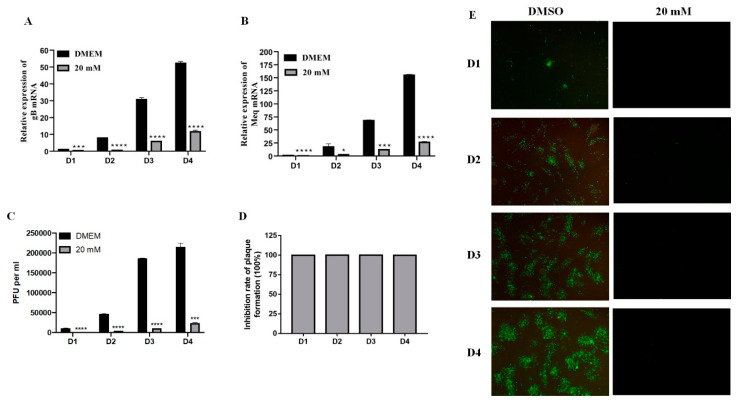
The inhibitory effect of LiCl on MDV replication in CEF is time-dependent. Total RNA was extracted from day 1 to day 4 after virus infection, and the expression levels of gB gene (**A**) and Meq gene (**B**) were detected by RT-qPCR. (**C**) Plaque count of MDV RB-1B strain infected CEF treated with 0 and 20 mM LiCl at different time points. (**D**) The inhibition rate of plaque was calculated and presented as mean plaque titers relative to the DMSO group. The mean plaque titer of the DMSO group was set to 100%. (**E**) Virus plaque formation was directly observed by indirect immunofluorescence assay at day 1 to day 4 after virus infection. The pictures were captured with an OLYMPUS microscope (×200). The data are expressed as the mean ± SD of three independent experiments, and analyzed by Student-t Test (* *p* < 0.05, *** *p* < 0.001, **** *p* < 0.0001) for A, B, and C.

Examination of gene expression in infected cells harvested at 24, 48, 72, and 96 h after 1000 pfu virus infection revealed that the inhibitory effect could be observed continuously at different time points post single treatment with 20 mM LiCl. As shown in Figure 2A,B, the significant inhibition of gB and Meq gene was observed from day 1 to day 4 post infection. The results of virus plaque count, the percentage of inhibition, and indirect immunofluorescence assay were also consistent with real-time PCR data (Figure 2C–E). Overall, these results indicated that 20 mM LiCl exhibits a significant inhibitory effect on MDV replication in a time-dependent manner in CEF cells.

### 2.3. Inhibitory Effect of LiCl Occurs throughout Virus Replication

**Figure 3:**
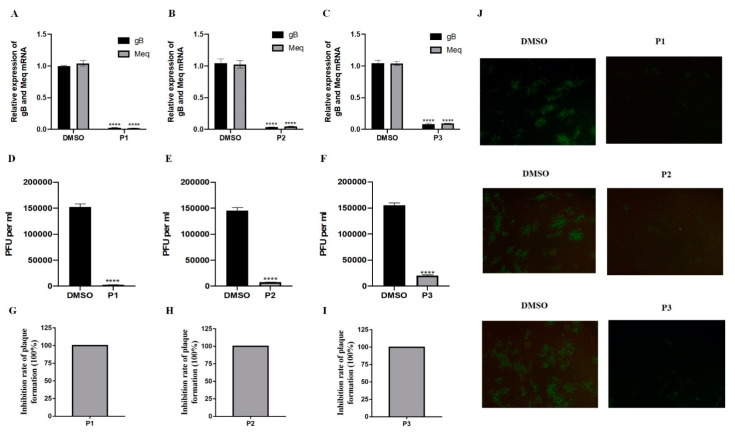
LiCl has an inhibitory effect in all stages of MDV infection. (**A**–**C**). The gene expression levels of Meq and gB were detected by RT-qPCR. The DMSO treated group was used as control. (**D**–**F**). Quantification of the virus by plaque counting. (**G**–**I**). The inhibition rate of plaque was calculated and presented as mean plaque titers relative to the DMSO group. The mean plaque titer of the DMSO group was set to 100%. (**J**). Detection of the inhibitory effect of LiCl on MDV RB-1B replication by indirect immunofluorescence assay. P1: cells were pretreated with 20 mM LiCl for 2 h before virus inoculation, P2: 20 mM LiCl and virus infected cells were added at the same time, and P3: 20 mM LiCl was added after virus inoculation. The pictures were captured with an OLYMPUS microscope (×200). The data are expressed as the average ± SD of three independent experiments, and analyzed by Student-t Test (**** *p* < 0.0001) for A and C.

In order to study the mechanism of LiCl inhibition on MDV infection, three time points related to the addition of LiCl (P1: before MDV (1000 pfu) infection, P2: during MDV infection, and P3: after MDV infection) were used, similar to our previous report [21]. The results of real-time PCR and plaque counts showed that the application of 20 mM LiCl had a significant inhibitory effect on MDV replication at all time points (Figure 3).

### 2.4. LiCl Affects the Infectivity of MDV Directly

**Figure 4:**
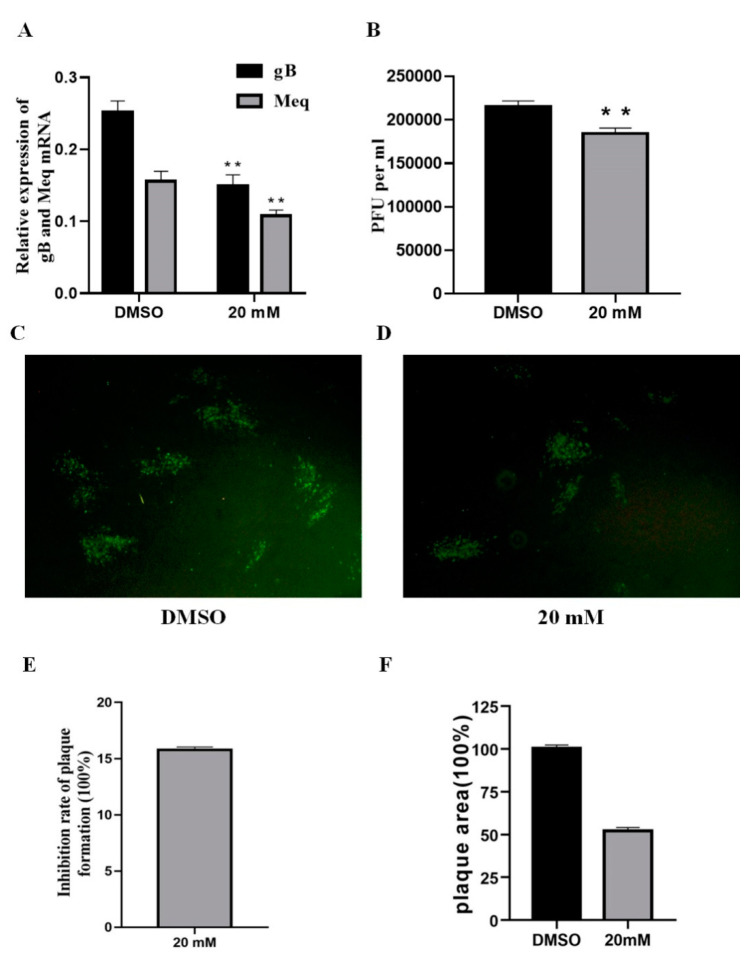
LiCl treatment pre-infection affects the infectivity of MDV. The mixture of 20 mM LiCl and MDV was incubated at 37 °C for 1.5 h, and then inoculated in CEF until harvesting. (**A**). Detection of Meq and gB transcript level by RT-qPCR from RNAs of infected CEF. (**B**). Virus titration by plaque counting. The inhibition was observed by indirect immunofluorescence assay in DMSO group (**C**) and 20 mM LiCl group (**D**). (**E**). Inhibition rate was calculated and presented as relative to the DMSO group. (**F**). Plaque sizes (30 plaques) were measured, and presented as mean plaque sizes relative to the DMSO group. The mean plaque size of the DMSO group was set to 100%. The error bar represents the standard deviation of three independent experiments. The pictures were captured with an OLYMPUS microscope (×200). The data are expressed as the mean ± SD from three independent experiments, and analyzed by Student-t Test (** *p* < 0.01) for A and B.

According to previous reports, LiCl can affect the infectivity of other viruses directly [22], thus we tried to explore whether LiCl had the same effect on MDV. The virus suspension was incubated with LiCl (20 mM) at 37 °C for 1.5 h, and then, CEF cells were infected for 96 h. The results of real-time PCR, virus titration, and indirect immunofluorescence showed that the virus gene expression and virus replication of the LiCl treated group were significantly reduced compared with the control group (Figure 4A–D). Similarly, inhibition rate and plaque area measurement results also indicated that LiCl could inhibit the infectivity of MDV directly (Figure 4E,F).

### 2.5. LiCl Inhibits Expression of Interferon-Related Gene in MDV Infected Cells

**Figure 5:**
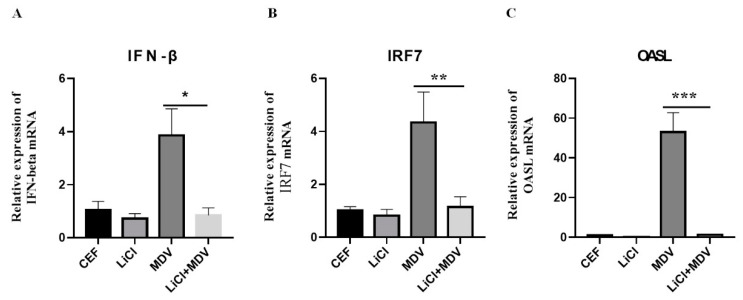
LiCl significantly inhibits the expression of interferon and related genes in MDV infected cells. (**A**). The expression of IFN-β in MDV-infected CEF cells after LiCl treatment. (**B**). The expression of IRF7 in MDV-infected CEF cells after LiCl treatment. (**C**). The expression of OASL in MDV-infected CEF cells after LiCl treatment. The data are expressed as the average ± SD of three independent experiments, and analyzed by Student-t Test (* *p* < 0.05, ** *p* < 0.01, *** *p* < 0.001).

LiCl can modulate the production of immune-related cytokines involved in the pathogenesis of various diseases [22,23,24]. In the current study, we investigated whether LiCl reduced the MDV replication by affecting the expression of interferon-related genes. We found that LiCl down regulated IFN-β, IRF7, and OASL gene expression in CEF cells infected with MDV (Figure 5).

## 3. Discussion

Vaccination with live attenuated vaccines is the major control strategy used against MD to prevent major economic losses associated with this important poultry disease worldwide. While MDV vaccines are relatively effective in protecting against clinical disease, vaccines are less effective in preventing infection and shedding of infectious virus [3]. This is thought to be one of the reasons for the continuous evolution of virulence and emergence of more virulent pathotypes [6]. Therefore, the development of anti-MDV drugs is of great significance for disease prevention and control.

In this study, we found that LiCl could significantly inhibit viral RNA and protein expression in CEF cells, and the antiviral effect of LiCl was time- and dose-dependent. The inhibitory effect on the replication and proliferation of MDV is similar to the research results reported previously on the activity of LiCl against herpes simplex virus [14,15,25], in which LiCl prevents viral replication by inhibiting viral DNA replication. In this study, we have shown that LiCl significantly inhibited viral gene expression, protein synthesis, and direct infectivity, and has an inhibitory effect at all stages of virus replication. These results strongly suggested that LiCl can have great potential for clinical application as an antiviral drug against MDV. Similar potential of LiCl against infectious bronchitis virus (IBV) has also been demonstrated [19]. Previous reports have shown that LiCl can inhibit virus replication by inhibiting virus-induced apoptosis, as has been shown with Coxsackie virus [26] and porcine epidemic diarrhea virus [18] infections, but without directly affecting virus invasion and entry into cells. Our results showing the effect of LiCl on the infectivity of MDV further demonstrate that LiCl functions through multiple antiviral mechanisms against different viruses. In this study, we believe that LiCl affects the infectivity of MDV through absorption by virus-infected CEF cells. MDV replication has been inhibited when the virus spread from the LiCl pre-treated inoculum to the CEF monolayer. However, we do not know the detailed mechanism, because how MDV gets in CEF cells is not fully understood.

In MDV-infected CEF cells, there was increased expression of IFN-β, IFN regulatory factor 7 (IRF7), and OASL as expected (Figure 5). In contrast, all of these gene expressions reduced significantly in LiCl treated cells. Similar results were also observed in the study of the antiviral effects of LiCl on coxsackie virus [26] and Sendai virus [27]. This result suggests that the anti-viral effect of LiCl on MDV might not depend on the expression of interferon and interferon-related factors. The specific mechanism needs further study for clarification.

All the experiments in this study were carried out in vitro, in CEF cells. At present, in vivo study has found that LiCl 20 mg/kg is not lethal to broilers and does not affect muscle mass. It can be used as a supplement that targets bone health. Therefore, LiCl has the potential to be used to improve bone formation and health quality of poultry [28]. Additional benefits of LiCl as an effective antiviral compound against MDV, potentially also reducing virus shedding and contamination of poultry house environment, can have a major impact on MD control that can work along with the current vaccination strategy.

In summary, this study shows that LiCl inhibits MDV replication and proliferation by inhibiting the expression of viral genes and proteins, in a dose- and time-dependent manner. In addition, LiCl can also directly affect the infectivity of the virus. These results indicate that LiCl can be used as a potential drug to inhibit MDV infection. Future studies will focus on the detailed mechanism of LiCl on MDV replication, and the antiviral effects of LiCl in chickens against MDV infection.

## 4. Materials and Methods

### 4.1. Viruses, Cells and Reagents

The RB-1B strain of MDV is cultivated and preserved in our laboratory. Nine-day old SPF chicken embryos (purchased from Lihua, Zhejiang, China) were used to prepare chicken embryo fibroblasts (CEF) for in vitro MDV culture. Fetal bovine serum (FBS), DMEM and 0.25% trypsin were purchased from GIBCO (Shanghai, China). The specific monoclonal antibody BA4 against MDV gB was generated and stored by our laboratory. FITC goat anti-mouse secondary antibody and LiCl was purchased from SIGMA (Shanghai, China). LiCl was dissolved with DMSO to a storage concentration of 20 M. HRP-enhanced chemical substrate and pre-stained protein molecular weight makers were purchased from Bio-Rad (Shanghai, China).

### 4.2. Virus Infection and LiCl Treatment

CEF cells were seeded in a 12-well plate (6.0 × 10^5^ cells/well) and maintained at 37 °C with 5.0% serum in DMEM. After treatment with 0, 2, or 20 mM LiCl for 2 h, the cells were infected with 1000 PFU of MDV (10 passages) and incubated for 96 h in the presence of LiCl. The real-time PCR, Western blotting, virus titration, and indirect immunofluorescence assays were carried out.

In order to determine the time dependent antiviral effect of LiCl, CEF cells in a 12-well plate were pretreated with LiCl (20 mM) for 12 h, and then each well was inoculated with 1000 PFU of MDV. After virus attachment for 4 h, the medium was replaced with a DMEM maintenance solution containing LiCl (20 mM) and 0.5% serum. Virus-infected cells were harvested on days 1, 2, 3, and 4 post-infection for plaque counting, real-time quantitative PCR detection and indirect immunofluorescence assay.

### 4.3. The Effect of Different Models of Drug Treatment

In order to study the mechanisms of LiCl inhibition of the virus, experiments related to the time of addition of LiCl were carried out as described previously [21]. Briefly, in the first experimental protocol (P1), CEF cells were pretreated with LiCl (20 mM) at 37 °C for 2 h, and the medium containing LiCl was removed before inoculation of 1000 PFU of virus. Infected CEF cells were then incubated at 37 °C and 5% CO2 in maintenance medium containing 0.5% FBS until 96 h post infection to allow MDV replication; in the second protocol (P2), a mixture of 1000 PFU of virus suspension and LiCl (20 mM) were added to the CEF cells and incubated at 37 °C for 6 h. After removing the medium mixture, the cells were incubated with the maintenance medium at 37 °C in 5% CO2 until harvesting; in the third protocol (P3), after the inoculum was attached with CEF in an incubator for 6 h, LiCl (20 mM) was added to the CEF cells in maintenance medium containing 0.5% FBS. After 96 h of incubation, all cells in 3 protocols were harvested for determining MDV plaque numbers and analysis of viral gene expression.

### 4.4. Evaluation of Virucidal Effect of LiCl on MDV

Similar to the protocols described in our previous report [21], 1000 PFU of virus stock was directly mixed with 20 mM LiCl in a tube and incubated at 37 °C for 1.5 h. After centrifugation to remove the supernatant, the virus-infected cells were resuspended and added to the CEF in a 12-well plate. After 4 h of virus attachment, the inoculum was replaced with fresh maintenance medium containing 0.5% serum. After 96 h of infection, cells were collected for virus titration and real-time PCR.

### 4.5. Virus Titrations and Plaque Area Determinations

As described in our previous report [21], trypsinised primary CEF cells were pated into each well of 96-well plate. The next day, 10-fold dilutions of the virus-containing cell suspension were added to the CEF, with 12 replicates of each dilution. The number of virus plaques was counted after 96 h. The highest dilution with plaques in all 12 wells was used for titer calculations using the formula: virus titer (PFU/mL) = (average number of plaques per well × dilution factor)/inoculated virus volume per well (mL). Plaque areas were measured from 30 selected plaques of each groups by Image J software, as described previously [29].

### 4.6. Real-Time PCR for Viral Gene Expression

As previously reported [30], the expression level of viral genes and cytokines was determined by 7500 Real-Time PCR System (ABI, Shanghai, China). The sequences of the primers are listed in Table 1, and these primer pairs were synthesized by Gene Script Company (Nanjing, China).

AxyPrep Multisource Total RNA Miniprep Kit (AXYGEN, Hangzhou, China) was used to prepare total RNA from CEF cells; 1 μg RNA was reverse transcribed into cDNA using PrimeScript RT Master Mix (TaKaRa, Dalian, China). Real-time fluorescent quantitative PCR (7500Real-Time PCR System, ABI) was used to detect the expression level of viral genes. The diluted cDNA (1 μL), 400 nM primers and 10 μL SYBR Green Master Mix were used for real-time PCR, and the reaction volume was 20 μL. The amplification conditions were as follows: 95 °C for 30 s, then 40 cycles, 95 °C for 5 s, and 60 °C for 34 s. After 40 cycles, a dissociation curve was generated to analyze each PCR product. The 2-^ΔΔ^CT method was used to analyze relative gene expression data with chicken 18S as an internal reference gene.

For detecting MDV genome quantity, the AxyPrep genomic DNA mini kit (AXYGEN, Hangzhou, China) was used to extract DNA, 100 ng DNA was used as the template, and q-PCR was used for the genome quantity of MDV DNA.4.7. Indirect immunofluorescence assay.

Infected cells were fixed with acetone ethanol (3:2) for 5–10 min at room temperature. After washing the cells with PBS for 3 times, the MDV-specific monoclonal antibody BA4 (10 μg/mL) was added and incubated in a 37 °C water bath for 1 h. After incubating with FITC-labelled goat anti-mouse secondary antibody for 30 min, images of plaques were captured with an OLYMPUS fluorescence microscope.

### 4.7. Western Blot Analysis

As described in our previous report [21], the protein concentrations of cellular proteins in the NP-40 cell lysate were determined using the BCA Protein Assay Kit (Bio-Rad, Shanghai, China).

### 4.8. Statistical Analysis

The results represented the mean standard deviation (SD) of three determinations. The GraphPad (version 8.0) software was used to analyze the differences in variability between experiments. The difference between two groups was evaluated by Student-t Test analysis. (Criterion: the difference of *p* value < 0.05 is statistically significant). The experiment was carried out at least three times independently.

## Figures and Tables

**Table 1:** Real-time PCR primer pairs.

Target Gene	Sequence	Product Size	Accession Number
Meq	F 5′-GTCCCCCCTCGATCTTTCTC-3′ R 5′-CGTCTGCTTCCTGCGTCTTC-3′	184	NC-002229.3
gB	F 5′-ACCCCATTCGGTGGCTTTTC-3′ R 5′-GCGTCCAGTTGTCTGAGG-3′	122	NC-002229.3
IRF7	F 5′-CGTATCTTCCGCATCCCTTGG-3′ R 5′-TCGTCGTTGCACTTGGAGCG-3′	206	NM-205372.1
IFN-β	F 5′-GCTCTCACCACCACCTTCTC-3′ R 5′-GCTTGCTTCTTGTCCTTGCT-3′	151	NM-001024836.1
OASL	F 5′-GAGATAGAGAAGGAGTGGTG-3′ R 5′-GTAGACTGTGGTCTTGTTAC-3′	201	NM_205041.2
18S	F 5′-TCAGATACCGTCGTAGTTCC-3′ R 5′-TTCCGTCAATTCCTTTAAGTT-3′	154	AF173612
OVO	F 5′-CACTGCCACTGGGCTCTGT-3′ R 5′-GCAATGGCAATAAACCTCCAA-3′	71	NM_205304.1
